# Adaptation of *Vibrio cholerae* to Hypoxic Environments

**DOI:** 10.3389/fmicb.2020.00739

**Published:** 2020-04-29

**Authors:** Emilio Bueno, Víctor Pinedo, Felipe Cava

**Affiliations:** Laboratory for Molecular Infection Medicine Sweden, Department of Molecular Biology, Umeå Centre for Microbial Research, Umeå University, Umeå, Sweden

**Keywords:** *Vibrio cholerae*, enteropathogen, respiration, nitrate, fumarate, TMAO, fermentation, fitness

## Abstract

Bacteria can colonize virtually any environment on Earth due to their remarkable capacity to detect and respond quickly and adequately to environmental stressors. *Vibrio cholerae* is a cosmopolitan bacterium that inhabits a vast range of environments. The *V. cholerae* life cycle comprises diverse environmental and infective stages. The bacterium is found in aquatic ecosystems both under free-living conditions or associated with a wide range of aquatic organisms, and some strains are also capable of causing epidemics in humans. In order to adapt between environments, *V. cholerae* possesses a versatile metabolism characterized by the rapid cross-regulation of energy-producing pathways. Low oxygen concentration is a key environmental factor that governs *V. cholerae* physiology. This article reviews the metabolic plasticity that enables *V. cholerae* to thrive on low oxygen concentrations and its role in environmental and host adaptation.

## Introduction

*Vibrio cholerae* is a Gram-negative facultative anaerobic bacterium that inhabits estuaries, rivers, and other aquatic environments (Reen et al., 2006) and can cause Cholera disease via contaminated water or food. Upon ingestion, the organism colonizes the small intestine (SI), where it secretes the potent cholera toxin (CT). CT is directly responsible for a purging diarrheal illness that can kill an adult in 24 h (Mekalanos et al., 1997). Cholera disease is also dependent on the production of the type IV pilus TCP (Waldor and Mekalanos, 1996; Häse and Mekalanos, 1998), the type VI secretion system T6SS (Fu et al., 2018), and alternative secreted virulence factors including the hemagglutinin/protease (HAP), the multifunctional RTX toxin, and the hemolysin A/cytolysin (VCC) (Hanne and Finkelstein, 1982; Olivier et al., 2007). During its infection, *V. cholerae* cells are shed in large numbers into the environment through the stool, where they can survive either as free-living cells, by forming biofilms on the chitin surface of crustaceans (Silva and Benitez, 2016) or by colonizing the gut of birds and fish until they are ingested again by humans, thus completing its life cycle (De Haan and Hirst, 2004; Yahr, 2006).

**FIGURE 1:**
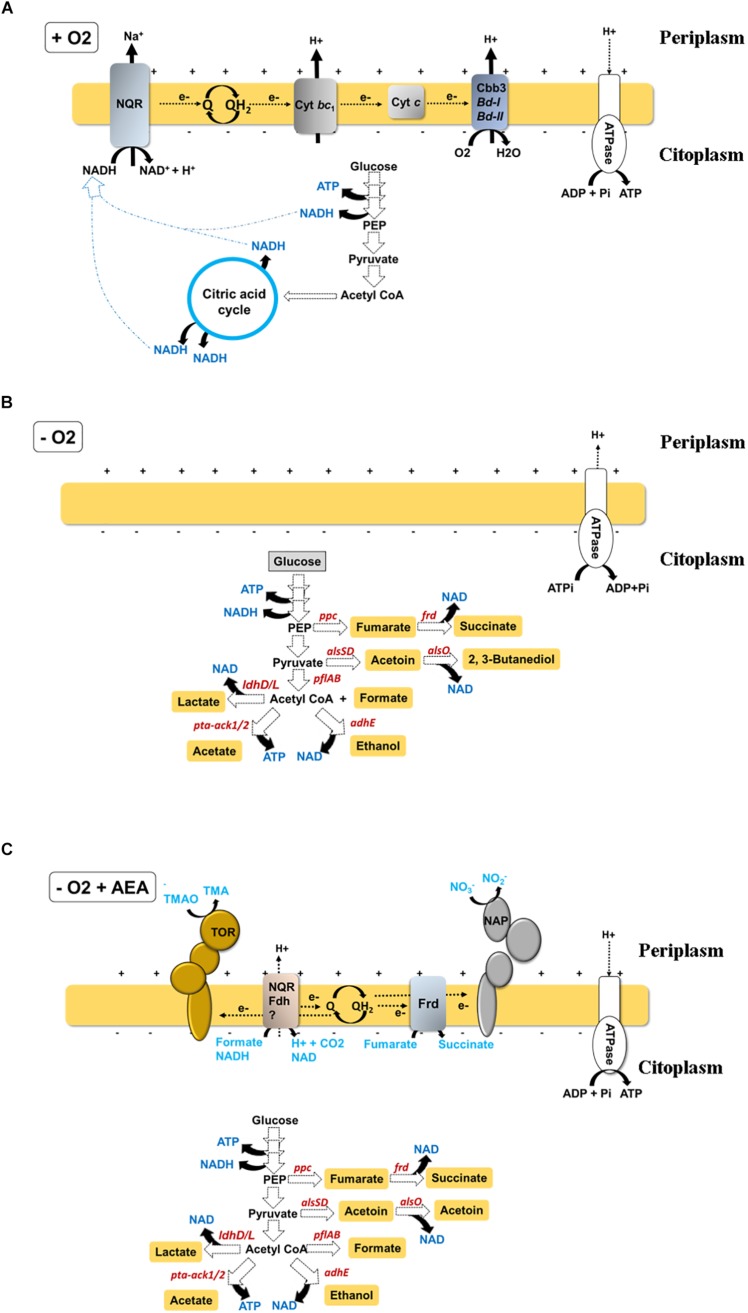
Schematic of redox balance and energy generating pathways in *V. cholerae* during **(A)** oxic growth, where *V. cholerae* obtains ATP through oxidative phosphorylation by respiration of oxygen using an electron transport chain initiated by a Na^+^-translocating NADH:quinone oxidoreductase (Na^+^-NQR), **(B)** hypoxic growth, where *V. cholerae* obtains energy by fermentation. In this condition, ATP is generated by substrate-level phosphorylation. As in the absence of final electron acceptors respiration is inhibited, the proton motive force (PMF) is established by proton pumping by the ATPase (with ATP consumption), and by sodium transporters **(C)** hypoxic growth in the presence of AEA, where *V. cholerae* is able to simultaneously obtain energy by substrate-level phosphorylation during fermentation and respiration of AEA. Represented in each scheme are only components experimentally demonstrated as relevant for the growth of the bacteria. Other components of the respiratory chain, such as alternative NADH dehydrogenases (see Table 1) whose inactivation does not affect *V. cholerae* growth are not shown.

These transitions of *V. cholerae* from the human gastrointestinal tract (GI) to the environment require a rapid metabolic adaption to a variety of environmental factors, adaptation to varying oxygen concentrations being one of the most significant. The most important requirement of any living organism is to respire oxygen, since its respiration (reduction to water) is the main source of energy for the cell. During aerobic respiration, electron donors such as NAD(P)H or FADH_2_ are oxidized, and the resulting free electrons are conducted through an electron transport chain (ETC) to the final electron acceptor, oxygen (Figure 1A). ETCs are formed by electron carriers with increasing redox potential embedded within cellular membranes. Typically, these electron transporters are transmembrane proteins, small mobile haem-containing proteins (cytochromes), and redox-active lipids (ubiquinones). The transport of electrons releases free energy that promotes translocation of protons through the membrane, generating an electrochemical gradient (aka. proton motive force, PMF). Dissipation of this proton gradient by an ATP synthase supports phosphorylation of ADP to render energy in the form of ATP. This process is commonly known as oxidative phosphorylation.

Although atmospheric oxygen concentration is 20.9%, partial pressure of oxygen in water drops to approximately 1% and fluctuates depending on the respiratory activity of neighboring organisms and the relative distance to the aerial surface (Mortimer, 1981). Furthermore, within the human small intestine, oxygen concentrations descend below 3% (Crompton et al., 1965; He et al., 1999) and are further lowered to a nearly anoxic environment by the action of the commensal microbiota and host metabolism (Byndloss et al., 2017).

To adapt to these shifts in oxygen concentrations, *V. cholerae* encodes a repertoire of four respiratory oxygen reductases (Heidelberg et al., 2000): three *bd*-type oxygen reductases that receive electrons directly from the ubiquinol pool (Yang et al., 2008) and one *cbb*_3_-type haem-copper oxygen reductase (Hemp et al., 2005) that receives electrons from the membrane-bound *bc*_1_ complex. These enzymes display a distinct affinity for oxygen (Esposti et al., 2019); however, biochemical studies on these respiratory complexes in *V. cholerae* are lacking.

In the total absence of oxygen as electron acceptor, *V. cholerae* can also grow by respiring (i.e., reducing) a variety of organic and inorganic alternative electron acceptors (AEA), including fumarate, nitrate (NO_3_^–^) (Bueno et al., 2018), and trimethylamine N-oxide (TMAO) (Braun and Thöny-Meyer, 2005; Lee et al., 2012). In niches where both O_2_ and an AEA are absent, *V. cholerae* has the capacity to grow fermenting diverse carbohydrates such as sucrose, dextrin, maltose, glucose, mannitol, sorbitol, lactose, and starch (Nobechi, 1925; Wang et al., 2009). Fermentation in *V. cholerae* varies depending on the strain. El Tor N16961 has the capacity to produce 2,3-butanediol as a fermentative neutral end product, avoiding acidification of the medium. In contrast, the classical biotype O395 is unable to synthesize 2,3-butanediol, and hence its viability is compromised during glucose mixed fermentation due to acidification of the medium by synthesis of organic acids (Yoon and Mekalanos, 2006; Lee et al., 2020).

Despite the importance of hypoxic metabolism in human pathogens, experimental studies in laboratories are still carried out in the presence of oxygen, conditions that pathogens will never face. In this review we dissect the metabolic pathways employed by *V. cholerae* to prevail when oxygen is scarce, with an emphasis on infective processes.

## Adaptations of *V. Cholerae* to Hypoxia

Under standard experimental conditions (i.e., aeration, 37°C, in LB medium), *V. cholerae* divides roughly every 16–20 min, yielding around 3 × 10^9^ CFU after 12 h of growth. Under these conditions, *V. cholerae* cells obtain energy in the form of ATP by respiration of oxygen, as eukaryotic mitochondria do (Figure 1A). However, in contrast to eukaryotic cells, *V. cholerae* is also able to generate energy and maintain its physiological functions in the absence of oxygen using AEA such as TMAO, fumarate and nitrate (Braun and Thöny-Meyer, 2005; Lee et al., 2012; Bueno et al., 2018; Figure 1C). In contrast to other enteric pathogens, *V. cholerae* cannot obtain energy to grow under hypoxia using DMSO (Braun and Thöny-Meyer, 2005; Lee et al., 2012), tetrathionate, or sulfate as AEA. In the absence of an AEA, *V. cholerae* still can survive by fermenting internal metabolic electron acceptors (EAs) derived from carbohydrate catabolism, such as pyruvate and acetyl coenzyme A (AcCoA) (Nobechi, 1924; Figure 1B).

In general, there is a hierarchy in the use of AEA, where the most efficient AEAs are reduced (NO_3_^–^ and TMAO) followed by those that yield less energy (nitrite, DMSO, tetrathionate). Ultimately, in the absence of AEAs, redox reactions are balanced by fermentation, where energy is generated by substrate-level phosphorylation (Unden and Bongaerts, 1997).

### Anaerobic Nitrate Respiration

Nitrate is an inorganic ion which is abundant in the environment and the human diet (Lundberg et al., 2004; World Health Organization [WHO], 2011; Weitzberg and Lundberg, 2013). Its respiration by bacteria can generate nitrogen gas (N_2_) or ammonium as final products. When the final product is N_2_ this process is known as denitrification, and it is one of the more important processes in the nitrogen cycle since it returns fixed nitrogen to the atmosphere and thereby completes the cycle (Martinez-Espinosa et al., 2011). This reductive process occurs in four steps, beginning with the reduction of NO_3_^–^ to NO_2_^–^, followed by the sequential reduction to the intermediates NO, N_2_O and finally to N_2_. The enzymes involved in denitrification are nitrate-, nitrite-, nitric oxide-, and nitrous oxide reductases, encoded by *nar/nap*, *nir/nrf*, *nor*, and *nos* genes, respectively (Bueno et al., 2012) whose synthesis is highly coordinated since accumulation of some of the nitrogen oxide intermediates, such as nitrite and nitric oxide, is toxic for the bacterial cells. Contrary to the anaerobic respiration of other AEA (e.g., TMAO, fumarate), which use menaquinone (MQ) as linker between NADH dehydrogenase and the terminal reductases, denitrification uses ubiquinone (UQ) (E_0_′ = +100 mV) to transfer electrons to the nitrate reductase (E_0_′ = +433 mV) (Unden and Bongaerts, 1997; Table 1).

**TABLE 1:** Standard redox potentials (mV) for electrons acceptor and donor couples.

**Redox couple**	**E_0_′(mV)**
O_2_/H_2_O	+818
${\text{NO}}_{3}^{-}/{\mathrm{NO}}_{2}^{-}$	+433
${\text{NO}}_{2}^{-}/{\mathrm{NH}}_{4}^{+}$	+360
Ubiquinone/ubiquinol	+100
DMSO/DMS	+160
TMAO/TMA	+130
Fumarate/succinate	+33
Menaquinone/menaquinol	+74
NAD^+^/NADH	−320
H^+^/H_2_	−432
${\text{CO}}_{2}/{\text{HCO}}_{2}^{-}$	−480

Despite the fact that anaerobic respiration is preferred over fermentation by most microbes, some enteropathogens such as *Escherichia coli*, *Salmonella typhimurium*, *Citrobacter rodentium*, and *V. cholerae*, simultaneously perform fermentation and respiration of NO_3_^–^ (Bueno et al., 2018; Figure 1C). In this scenario, fermentative products may acidify the growth medium and protonate NO_2_^–^, the product of NO_3_^–^ respiration, to nitrous acid HNO_2_ (Da Silva et al., 2006) which might cross the bacterial membrane to interfere with diverse metabolic functions of the organism (Rowe et al., 1979). Additionally, HNO_2_ has been suggested to act as an uncoupler of the PMF (Meijer et al., 1979; Sijbesma et al., 1996) and generates a variety of reactive nitrogen species (RNS), including nitric oxide (NO), nitrogen dioxide (NO_2_), dinitrogen trioxide (N_2_O_3_), dinitrogen tetraoxide (N_2_O_4_), nitrite (NO_2_^–^), S-nitrosothiols, peroxinitrite (ONOO^–^) and nitrate. This repertoire of RNS may interact with numerous targets within microbial cells, including tyrosine residues of proteins, thiols and metal centers, and nucleotide and membrane lipids (Fang, 1997; Nathan and Shiloh, 2000), thereby inhibiting their biological functions.

**FIGURE 2:**
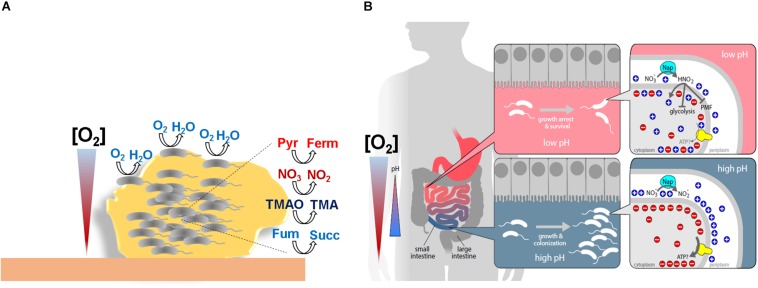
Schematic representations of *V. cholerae* niches where oxygen concentrations are limited. **(A)** Within the biofilm bacteria faces different oxygen concentrations. Cells situated in the periphery of the biofilm, where oxygen tensions are higher, will obtain energy through respiration of oxygen. However, cells situated in inner layers, where oxygen concentrations are scarce, will obtain energy through fermentation or/and nitrate, TMAO, fumarate respiration. **(B)** Human intestine colonization model showing the divergent outcomes during anaerobic nitrate respiration on bacterial expansion dependent on oxygen concentrations and pH. Pyr: pyruvate. Ferm: fermentative products. Fum: fumarate. Succ: succinate.

The denitrification pathway is generally conserved among enteric pathogens (Arkenberg et al., 2011) but not in *V. cholerae*, which, despite having a periplasmic nitrate reductase Nap operon (*vca0676-80*) lacks the nitrite (Nir/Nrf), nitric oxide (Nor), or nitrous oxide (Nos) reductases (Heidelberg et al., 2000). As a consequence, *V. cholerae* accumulates toxic nitrite in anoxia in the presence of nitrate and so it was believed that it was unable to grow by nitrate respiration (Braun and Thöny-Meyer, 2005). However, it has been demonstrated that this inability was due to membrane potential dissipation by the formation of nitrous acid under fermentative acidifying conditions. Thus, the capacity of *V. cholerae* to grow by nitrate reduction, as denitrifiers do, was achieved by buffering the pH of the growth medium, minimizing the production of nitrous acid (Bueno et al., 2018). Although in microbiology the capacity to grow is always considered a positive trait, it was found that the growth arrest exerted by nitrite accumulation under low pH also had a relevant function for *V. cholerae* and other enteric pathogens, as it lead to increased cell viability during fermentative acidification of the media (Bueno et al., 2018; Figure 2B). Since nitrite accumulation is widespread among nitrate reducing bacterial species (Almeida et al., 1995; Bueno et al., 2008; Bergaust et al., 2010; Rowley et al., 2012), these results suggested an unprecedented role for nitrite in bacterial persistence.

Regulation of the reduction of NO_3_^–^ to NO_2_^–^ has been comprehensively studied in *E. coli*. The transcriptional control of this process has been attributed to three systems: (i) Fnr transcriptional factor (fumarate nitrate reductase regulator), and the two-component systems (TCS): (ii) NarX-NarL, and (iii) NarQ-NarP (Rabin and Stewart, 1992; Noriega et al., 2010). Fnr is the transcriptional factor implicated in sensing external O_2_ concentrations and inducing the expression of anoxic lifestyle genes., such as the fermentative and the nitrate reduction *nap/nar* genes (Melville and Gunsalus, 1996; Lamberg and Kiley, 2000; Stewart, 2003; Browning et al., 2004; Crack et al., 2004). While NarXL in *E. coli* is a specific system for nitrate response, NarQP can sense nitrate, nitrite, and other signals (Lee et al., 1999; Noriega et al., 2010). The presence in *V. cholerae* of a homologous system to the *E. coli* NarQP suggests that the overall responses to nitrate and nitrogen oxides intermediates derived from its reduction might be entirely governed by this two-component system.

### Anaerobic TMAO Respiration

TMAO is a small organic molecule abundant in the environment, aquatic animals and humans. In aquatic animals it supports homeostasis during changes in hydrostatic pressure and salinity (Pang et al., 1977; Zerbst-Boroffka et al., 2005). In humans, TMAO is mostly generated from trimethylamine (TMA) oxidation, which is a catabolic product of choline, carnitine, and phosphatidylcholine by the gut microbiota (Koeth et al., 2013).

When oxygen is scarce, TMAO can substitute for oxygen as the final electron acceptor in the *V. cholerae* respiratory chain to produce energy during its reduction to TMA (Figure 1C) by the TMAO reductase complex. TMAO reductase is encoded by the *tor* operon and consists of TorA, the TMAO reductase enzyme; TorC, a *c*-type cytochrome; and TorD, a specific chaperone for TorA (Silvestro et al., 1989; Pommier et al., 1998; Ansaldi et al., 1999). Since the redox potential of TMAO is lower than that of NO_3_^–^ (E_0_′ = +130 mV vs +433 mV), menaquinone (E_0_′ = −74 mV), a quinone with lower redox potential, replaces ubiquinone as the adapter molecule for the electron transfer to TMAO (Table 1). Interestingly, aerobic expression of Tor in *E. coli* was demonstrated to be a stochastic bet-hedging strategy to avoid sudden anoxic entrapment (Carey et al., 2018). Whether such regulation of Tor is also present in *V. cholerae* is currently unknown.

One unique feature of anaerobic TMAO respiration in *V. cholerae* is that, while the presence of TMAO supports a rapid increase of biomass during the first 4 h of incubation, cell viability is largely impaired by a ppGpp-dependent response once stationary phase is reached (Oh et al., 2014). Interestingly, cholera toxin (CT) is produced during TMAO respiration (Childers and Klose, 2007; Lee et al., 2012), however, the CT inducer does not seem to be the respiration of TMAO itself but rather an intermediate product such as ROS followed by the ppGpp-dependent stringent response (Lee et al., 2012; Oh et al., 2014). In fact, addition of H_2_O_2_ in the presence of TMAO also enhanced the synthesis of CT. These results might suggest that TMAO-dependent CT production in *V. cholerae* may be linked with the stress conditions experienced by the pathogen while colonizing the host and thus it might aid the development of the infection.

Expression of the *torCAD* operon is controlled by a three-component system composed by the proteins TorT, TorR and TorS (Baraquet et al., 2006; Moore and Hendrickson, 2012). When there is TMAO in the medium, the periplasmic sensor TorT interacts with the membrane-bound protein TorS and this phosphorylates TorR, which ultimately induces the expression of *torCAD*. In contrast to other alternative respiratory systems, expression of the Tor system is not induced by the absence of oxygen in *E. coli* (Ansaldi et al., 2007) and it is independent from the global hypoxia regulators (i.e., FNR and ArcBA) (Pascal et al., 1984; Lynch and Lin, 1996) or from AEA signals such as nitrate (Pascal et al., 1984; Ansaldi et al., 2007). In addition to TorSR, TMAO respiration in *V. cholerae* is also governed by the catabolite repression protein (CRP), impairing TMAO reduction when glucose is available under anaerobic conditions. It has been proposed that the depletion of intracellular cAMP under high glucose concentrations leads to the inactivation of CRP and, as a consequence, expression of the TMAO reductase TorA is abrogated (Oh et al., 2015).

Despite that dimethyl sulfoxide (DMSO) is an organic compound analogous to TMAO, and it is also serves as final electron acceptor in some bacterial species (McCrindle et al., 2005), *V. cholerae* is unable to use it for anaerobic respiration (Braun and Thöny-Meyer, 2005; Lee et al., 2012).

### Anaerobic Fumarate Respiration

Fumarate is an intermediate in the tricarboxylic acid (TCA) cycle, which can also replace the role of oxygen as respiratory final electron acceptor in *V. cholerae* (Figure 1C; Braun and Thöny-Meyer, 2005; Lee et al., 2012). Although fumarate presents a very low redox potential (E_0_′ = 30 mV), it supports robust anaerobic growth in *V. cholerae* (Braun and Thöny-Meyer, 2005; Lee et al., 2012) and as for TMAO respiration, menaquinone is used as the ETC adaptor for fumarate (Table 1). In aerobiosis, fumarate is generated from succinate by the TCA cycle enzyme succinate dehydrogenase (Sdh), but in the absence of oxygen, the reverse reaction can be catalyzed by the fumarate reductase (Frd), hence allowing the resulting fumarate to be used as the AEA.

The fumarate reductase in *V. cholerae* is a membrane-bound complex composed of four polypeptides designated FrdA, FrdB, FrdC, and FrdD (VC2656-2659) (Heidelberg et al., 2000). The catalytic FrdAB components face the inner side of the cytoplasmic membrane, where fumarate is reduced to succinate (Lemire et al., 1982). Despite being the most extensive form of anaerobic respiration, regulation of fumarate respiration in *V. cholerae*, and its importance for colonization during infection, is not yet known. In *E.coli*, FNR and Arc control the expression of the *frdABCD* operon during hypoxic conditions (Jones and Gunsalus, 1987; Iuchi and Lin, 1988; Park et al., 1995) but if NO_3_^–^ is present in the environment, expression of the *frd* operon is suppressed by the Nar two-component regulatory system (Gunsalus, 1992). Therefore, the response to nitrate is dominant over anoxia in modulating the expression of the fumarate respiration machinery (Jones and Gunsalus, 1985, Gunsalus, 1987). In addition, exogenous fumarate also regulates the *frdABCD* operon via the DcuSR regulatory two-component system (Zientz et al., 1998). Since TMAO does not appear to control fumarate reductase gene expression it was suggested that *E. coli* does not display a hierarchical use of TMAO over fumarate (Jones and Gunsalus, 1987).

### Fermentative Metabolism

When both oxygen and an AEA are absent in the environment, *V. cholerae* is still able to generate energy and maintain its physiological functions by inducing the fermentative pathway (Figure 1B). In comparison to the AEA-dependent pathways, fermentation is the lowest energy generating pathway where energy is produced by substrate-level phosphorylation and pyridine nucleotides replace quinones as intermediate electron drivers.

Fermentation can be divided into two parts: first, glucose oxidation produces NADH and pyruvate, and second, pyruvate is reduced and NAD^+^ regenerated. Although there is only a one pathway for the first step, there are multiple alternatives for the second, which produces lactate, acetate, ethanol, formate and 2,3-Butanediol (Yoon and Mekalanos, 2006; Hawver et al., 2016; Bueno et al., 2018).

While reduction of pyruvate to lactate and ethanol provides a mechanism for NAD^+^ regeneration, the acetate branch generates energy in the form of ATP (Figure 1B). The lactate dehydrogenase enzyme (Ldh) catalyzes the reduction of pyruvate to lactate. Although inactivation of *E. coli* Ldh does not have any effect on hypoxic growth *in vitro* (Mat-Jan et al., 1989), the role of the *V. cholerae* lactate dehydrogenase LdhA is still unknown.

AcCoA is a vital metabolic intermediate for any living organism (Pietrocola et al., 2015). When oxygen is present, the so-called pyruvate dehydrogenase complex (PDC) transforms pyruvate to AcCoA with the generation of NADH, however, under hypoxia, the high NADH/NAD^+^ ratio inactivates the PDH complex (Hansen and Henning, 1966). Under these conditions, pyruvate will then be converted to AcCoA by the oxygen sensitive PflAB pyruvate formate-lyase complex (PFL), with the co-production of formate, which is a common electron donor during anaerobic nitrate respiration and the substrate for dihydrogen production (Cole and Wimpenny, 1968; Garland et al., 1975). In contrast to the oxidation of pyruvate to AcCoA in the presence of oxygen, AcCoA generation under anaerobiosis does not generate reducing equivalents. Thus, formate production during fermentation is the best suited reaction for balancing cellular redox status. Given its toxicity, formate is rapidly pumped out into the bacterial periplasm by the transporter FocA where it is used as electron donor by the FDH-N or FDH-O formate dehydrogenases for nitrate or oxygen respiration, respectively. This process is coupled with the generation of PMF (Clark, 1989; Sawers, 2005). Under hypoxic conditions, inactivation of PFL in *E. coli* reduces energy production and as consequence, the bacterial growth yield is impaired. The bacterium is able to counter this defect by the coupled induction of the glycolytic glyceraldehyde 3-phosphate dehydrogenase (GAPDH) and lactate dehydrogenase, to increase cellular ATP and dissipate the excess of NADH produced by GAPDH, respectively (Zhu and Shimizu, 2004). The importance of this pathway in the hypoxic metabolism of *V. cholerae* also remains unknown.

The second NAD^+^ regenerating pathway consists of the reduction of AcCoA to acetaldehyde and this to ethanol by the alcohol dehydrogenase (AdhE) protein (Clark, 1989). Inactivation of AdhE in *V. cholerae* renders strains incapable of growing under fermentative conditions with glucose as the sole carbon source (Bueno et al., 2018), suggesting that NAD^+^ regeneration cannot be compensated by other NAD^+^ producing branches such as reduction of pyruvate to lactate. Interestingly, addition of an external electron acceptor such as nitrate relieves the redox and energetic constraints supporting *V. cholerae* growth (Bueno et al., 2018).

Acetate is generated in two steps, AcCoA produced in glycolysis is converted to acetyl-phosphate by the phosphotransferase PTA, and subsequently, the acetate kinase Ack generates acetate. Inactivation of this fermentative branch in *E. coli* provokes a remarkable perturbation of the bacterial fermentative profile under hypoxia which results in impaired bacterial growth rates and synthesis of fermentative intermediates, which is countered by lactate overflow (Castaño-Cerezo et al., 2009). In contrast to *E. coli*, *V. cholerae* presents two copies of *ack*, *ack*_1_, and *ack*_2_. Despite that the function of Ack remains unknown in *V. cholerae*, this seeming functional redundancy suggests the particular importance that this fermentative branch might have for the hypoxic lifestyle in *V. cholerae*.

2,3-Butanediol is produced by *V. cholerae* as well as by a few enterobacteria such as *Serratia*, *Klebsiella*, or *Enterobacter* species (White, 2000). Production of this compound benefits the cell since it decreases the production of fermentative organic acids, thus reducing the otherwise detrimental acidification of the medium. In *V. cholerae*, fermentation varies depending on the strain. While the El Tor biotype strain N16961 strain produces 2,3-butanediol, the classical biotype strain O395 is unable to synthesize this compound, and as a result its viability is compromised during glucose mixed fermentation (Yoon and Mekalanos, 2006). Interestingly, although *V. cholerae* biotype strain N16961 synthesizes 2,3-butanediol (Yoon and Mekalanos, 2006), we have observed that this compound does not control the drop of pH due to fermentation under strict anaerobic conditions in the biotype strain C6706 (Bueno et al., 2018). The presence of oxygen in the mentioned studies (Yoon and Mekalanos, 2006) might suggest that 2,3-Butanediol production in *V. cholerae* is reduced under hypoxic conditions, suggesting potential benefits of accumulating fermentative organic acids during anaerobic respiration.

In *E. coli*, fermentative metabolism is controlled by the FNR (fumarate, nitrate respiration) transcriptional regulator (Gunsalus, 1992; Becker et al., 1996) and the ArcBA (aerobic respiration control) two-component regulatory system (Spiro and Guest, 1991; Sawers and Suppmann, 1992; Gunsalus and Park, 1994; Guest et al., 1996; Georgellis et al., 1999; Levanon et al., 2005; Shalel-Levanon et al., 2005; Unden et al., 1995). However, expression of the *adhE* gene is governed by the Fis (Factor for inversion stimulation) and Cra (Catabolite repressor activator) proteins and the cellular NADH/NAD^+^ ratio (Membrillo-Hernández and Lin, 1999). The presence of homologous systems to FNR/ArcBA in *V. cholerae* might suggest their implication in regulating fermentative metabolism, however, their relevance for hypoxic metabolism is still unknown.

Despite the evident benefits of fermentative metabolism in facultative anaerobes, one drawback is the acidification of the growth medium by the production of organic acids. Fermentative enterobacteria, and especially *V. cholerae*, grow optimally at an alkaline pH. Hence, it was speculated that fermentative acidification to pH 4,5 impaired bacterial growth rate and viability. Surprisingly, we have found that fermentative acidification combined with nitrite production drives *V. cholerae* into a growth-arrested persistent mode. Interestingly, this survival mechanism is spread among diverse enteric pathogens underscoring a potential strategy to adapt to the hypoxic intestine during infection (Bueno et al., 2018). From an ecological and evolutionary point of view, these findings provide insights into why some bacteria retain certain processes that in principle can be considered undesirable under certain conditions. In addition to the role of toxic products such as nitrous acid during fermentative acidification and nitrate reduction, they could also be used by bacteria as metabolic weapons against their hosts during infection.

## Relevance of Hypoxic Metabolism in *V. Cholerae* Niches

The life cycle of *V. cholerae* includes infective and environmental stages where *V. cholerae* accumulates and forms polymicrobial aggregates such as sludge, films, mats, flocs, or biofilms (Faruque et al., 2006; Islam et al., 2007; Tamayo et al., 2010). Biofilms present a dense structure that together with high bacterial respiratory activity results in an abrupt drop in their internal oxygen concentration (Xu et al., 1998; Walters et al., 2003; Werner et al., 2004; Jo et al., 2017; Karampatzakis et al., 2017). In addition to biofilms, *V. cholerae* faces low oxygen tensions and anoxia due to the oxidative metabolism of commensal microorganisms and host colonocytes in the human gut during infection (Zheng et al., 2015; Byndloss et al., 2017; Chun et al., 2017). Under such conditions, energy generation by canonical oxygen respiratory systems will not proceed and the bacterium must re-program its metabolism to obtain energy while keeping up its cellular redox status. *V. cholerae* might achieve this by (i) exchanging common oxygen respiratory terminal oxidases with oxidases that have higher affinity for oxygen (Heidelberg et al., 2000), (ii) using fermentative pathways, or (iii) using an AEA (Bueno et al., 2018; Figure 2A). Indeed, it has been demonstrated experimentally that biofilm formation in *Pseudomonas aeruginosa, Streptococcus aureus, Escherichia coli*, and *Bacillus subtilis* induces hypoxia related enzymes such as high-oxygen-affinity terminal oxidases (Jo et al., 2017), anaerobic ribonucleotide reductases (RNR) (Crespo et al., 2016), anaerobic threonine fermentation (Létoffé et al., 2017), mixed fermentative enzymes (Chen et al., 2015), and anaerobic nitrate respiration (Yoon et al., 2004), which are crucial for the development and maturation of the biofilm.

Although the relevance of hypoxic metabolism for *V. cholerae* biofilm development and turnover has not yet been investigated, induction of hypoxic related pathways in *V. cholerae* was identified by transcriptomic studies during colonization experiments using the mouse and rabbit infection models (Table 2; Merrell et al., 2002; Bina et al., 2003; Xu et al., 2003; Yoon and Mekalanos, 2006; Schild et al., 2007; Mandlik et al., 2011; Minato et al., 2014; Oh et al., 2014; Bueno et al., 2018).

**TABLE 2:** Determinants supporting *V. cholerae* energetic metabolism under oxic, hypoxic and hypoxic respiratory conditions.

**Energy generating enzymes**	**Redox couple**	**Relevance *in vitro***	**Expression or relevance *in vivo***
*Oxic related systems*			
NADH succinate dehydrogenase. *sdhABCD*	Succinate/fumarate	ND^1^	ND^6,7,8,9,10^
NADH dehydrogenase, putative. *vc1581-2*	NAD^+^/NADH	ND^1^	Induced in mouse^9^
NADH dehydrogenase. *ndh vc1890*	NAD^+^/NADH	ND^1^	Reduced in rabbit ileal loop^7^
NADH dehydrogenase, putative. *Vca0155*	NAD^+^/NADH	ND^1^	Induced in rabbit ^7,9^ and reduced in mouse^9^
D-amino acid dehydrogenase. *dadA*	D-aminoacid/2-oxoacid+${\text{NH}}_{4}^{+}$	ND^1^	ND^6,7,8,9,10^
Aerobic glycerol-3-phosphate dehydrogenase. *vca*	: DHAP/Gly-3-PO_4_	ND^1^	Induced in mouse^9^
NQR. *nqrFABC*	NAD^+^/NADH	Delayed growth in +O_2_ and −O_2_^2^	ND^6,7,8,9,10^
Cytochrome *d* ubiquinol oxidase. *cydAB-1*	O_2_/H_2_O	ND^1^	Reduced in mouse^9^
Cytochrome *d* ubiquinol oxidase. *cydAB-2*	O_2_/H_2_O	ND^1^	Induced in mouse^9^
Cytochrome *cbb*_3_ oxidase. *ccoNOQP*	O_2_/H_2_O	ND^1^	Induced in rabbit ileal loop^7^ and reduced in mouse^9^
*Hypoxic related systems*			
D-lactate dehydrogenase. *IdhA*	D-lactate/Pyruvate	ND^1^	ND^6,7,8,9,10^
L-lactate dehydrogenase. *lldD*	L-lactate/Pyruvate	ND^1^	Induced in mouse^9^ and rabbit^9^ and late infection in mouse^8^
Alcohol dehydrogenase/acetaldehyde dehydrogena	se. *ad*P*h*y*E*ruvate/Ethanol	Reduced growth in −O_2_^1^	Reduced in rabbit^9^ and induced in rabbit ileal loop^7^
Acetate kinase. *ack1/ack2*	Acetyl-phosphate/Acetate	Delayed growth in +O_2_ and −O_2_^1^	ND^6,7,8,9,10^
Phosphate transferase. *pta*	Acetyl-C oA/Acetyl-p h osph ate	ND^1^	ND^6,7,8,9,10^
Pyruvate formate lyase. *pflAB*	Pyruvate/Formate	Reduced growth in −O2 ^1^	ND^6,7,8,9,10^
Acetolactate decarboxylase/acetoin reductase. *als*	*S*P*D*y*O*ruvate/Acetoin-Butanediol	Reduced growth in −O2 ^5^	Reduced fitness *in vivo*^5^
*Hypoxic respiratory systems*			
Nitrate reductase. *nap*	${\text{NO}}_{3}^{-}/{\text{NO}}_{2}^{-}$	Growth defect in −O2 +NO3- ^3^	Increased in mouse^9^ and stools^6^. Reduced fitness *in vivo*^3^
Tmao reductase. *tor*	TMAO/TMA	Growth defect in −O2 +TMAO ^4^	Increased in mouse early infection^8^
Fumarate reductase. *frd*	Fumarate/Succinate	Growth defect in −O2 +Fumarate ^1^	Increased in mouse^9^, rabbit ileal loop^7^ and human stools^10^
Formate dehydrogenase. *fdhB-fdnI*	Formate/${\text{HO}}_{2}^{{}^{-}}$	ND^1^	Increased in rabbit^9^ and human stools^10^

*The enzymes substrates and its products are shown in the second column. The third column shows the *in vitro* phenotype after inactivation of each determinant in *V. cholerae* (ND = no differences detected). The fourth column compiles gene expression modulation in mouse or rabbit intestine during bacterial colonization, during its transit in the last stages of colonization and from stools of patients with the cholera disease. 1. Bueno et al., unpublished results. 2. Minato et al., 2014. 3. Bueno et al., 2018. 4. Oh et al., 2014. 5. Yoon and Mekalanos, 2006. 6. Merrell et al., 2002. 7. Xu et al., 2003. 8. Schild et al., 2007. 9. Mandlik et al., 2011. 10. Bina et al., 2003.*

Regarding fermentative metabolism, expression of genes encoding the fermentative enzymes L-lactate dehydrogenase (L-LdhA), acetolactate decarboxylase/acetoin reductase (AlsSDO) and alcohol dehydrogenase AdhE were induced during *V. cholerae* colonization (Table 2; Xu et al., 2003; Yoon and Mekalanos, 2006; Schild et al., 2007; Mandlik et al., 2011). The prominent induction of the two branches which dissipate redox potential during hypoxic growth through the generation of lactate and ethanol might suggest that during infection *V. cholerae* experiences a remarkable redox constraint, probably due to the low oxygen concentrations prevalent in the overpopulated intestines. The *als* operon, implicated in the synthesis of 2,3-butanediol, has a role in maintaining the pH at neutral levels during fermentative processes (Yoon and Mekalanos, 2006). Therefore, production 2,3-butanediol by certain *V. cholerae* strains (e.g., N16961) underscores the importance of countering the acidifying fermentative processes during hypoxic growth *in vivo*. Similarly to fermentative metabolism, bacterial respiratory pathways also seem to be modulated during infective processes. Thus, expression of *V. cholerae* respiratory enzymes such as nitrate (Nap), TMAO (Tor), and fumarate reductases (Frd) were also induced during infection experiments in mice, and consistently, inactivation of Nap and Tor respiratory systems reduced *V. cholerae* fitness during intestinal colonization in mice (Table 2; Oh et al., 2014; Bueno et al., 2018). Interestingly, expression of the nitrate reductase and fumarate reductases was also enhanced in human stool samples, suggesting a possible role of these respiratory enzymes in the dissemination and transmission of *V. cholerae* (Table 2; Merrell et al., 2002; Bina et al., 2003).

In addition to the activation of hypoxic metabolism to maintain bacterial energy when oxygen is scarce, low oxygen concentrations could be a niche specific signal (e.g., indicating a host niche), able to trigger virulence programs in pathogens. Indeed, induction of the toxin-coregulated pilus (TCP) was detected when *V. cholerae* cultures were subjected to oxygen deprivation (Marrero et al., 2009), suggesting that hypoxia might be a key signal used by the pathogen to determine niche localization. In addition, induction of TCP was also found to be dependent on low oxygen concentrations by reduction of the cysteine-containing regulatory protein OhrR in concert with AphB (Liu et al., 2011, 2016). Although anoxia itself seemed not to induce the synthesis of the CT in *V. cholerae* (Krishnan et al., 2004; Marrero et al., 2009), CT was highly stimulated by the presence of TMAO as final electron acceptor (Lee et al., 2012; Oh et al., 2014). Interestingly, TMAO respiration during host colonization has only been shown to be relevant in the enteropathogen *V. cholerae.*

The importance of hypoxic metabolism in infection has also been reported in other pathogenic bacteria. In *Brucella suis* for example, persistence within mouse macrophages is mediated by the induction of the high-oxygen-affinity terminal oxidase *cbb*_3_ (Jiménez De Bagüés et al., 2007). The fermentative pathway was also found to be induced in pathogenic bacteria during infection (Beckham et al., 2014; Luong et al., 2015). Similarly, the anaerobic respiration of nitrate (Winter et al., 2013; Lopez et al., 2015), tetrathionate (Winter et al., 2010), 1,2-propanediol, and fumarate (Ge et al., 2000; Jones et al., 2007) was induced during infection, and enhanced pathogen survival and persistence within the host.

Altogether, this experimental evidence suggests that hypoxic metabolism plays an important role in a variety of bacterial pathogens under both free-living conditions and during infective processes. However, whether induction of hypoxic metabolism is strictly only required for balancing bacterial energetics or if it has also a direct role in bacterial pathogenicity during infective stages (e.g., synthesis of bacterial effectors and toxins) still needs to be addressed.

## Conclusion and Future Perspectives

Human infectious diseases caused by bacterial pathogens remain a global concern for public health care systems which result in millions of deaths per year worldwide. Oxygen concentration is low in most tissues within the host. Therefore, the standard laboratory aerobic culture of bacteria is undoubtedly far from mimicking the real scenario within the host and will result in misleading findings in understanding the molecular mechanisms used by pathogenic bacteria to cause diseases. Thus, considering that the hypoxic metabolic state is the prevalent physiological condition of pathogens during infection, and adopting adequate methodologies for their studies is a must in order to solve new enigmas in host-pathogen interactions.

Despite our deep knowledge of microbial fermentative and respiratory pathways, a comprehensive understanding of the crosstalk among hypoxic processes is still missing. In the environment or within the host, several types of fermentable carbon sources and respiratory terminal acceptors are simultaneously available, and hence, understanding pathogen responses while co-metabolizing different hypoxic substrates is an important question that remains to be addressed. In addition, with the advent of novel microscopy techniques, flow cytometry and microfluidic devices it will be possible to visualize the dynamics of those systems in real-time.

Regardless of the importance of studying the behavior of clonal bacterial populations, it has been demonstrated that small sub-populations can present altered behavior within the community (Veening et al., 2008; Lycus et al., 2018). Thus, understanding hypoxic regulation at single cell level represents another missing piece in better understanding population responses. In fact, in nature there are multiple cases of metabolic cooperation and competition between organisms co-inhabiting a niche. However, how hypoxic metabolic products influence or modulate the host’s microbiota and immune system during infection is unknown. Hence, given that many enteropathogens accumulate nitrite, it is possible that nitrite may be instrumental for pathogenic bacteria to compete against neighboring commensals and/or modulate the host immune system during infection. Future research combining omic data with single cell mechanistic insights will shed light on how regulation of hypoxic metabolism is orchestrated both *in vitro* and *in vivo*.

## Author Contributions

All authors listed have made a substantial, direct and intellectual contribution to the work, and approved it for publication.

## Conflict of Interest

The authors declare that the research was conducted in the absence of any commercial or financial relationships that could be construed as a potential conflict of interest.
